# Some inequalities for Cesàro means of double Vilenkin–Fourier series

**DOI:** 10.1186/s13660-018-1929-y

**Published:** 2018-12-19

**Authors:** T. Tepnadze, L. E. Persson

**Affiliations:** The Artic University of Norway, Narvik, Norway

**Keywords:** 42C10, 42B25, Inequalities, Approximation, Vilenkin system, Vilenkin–Fourier series, Cesàro means, Convergence in norm

## Abstract

In this paper, we state and prove some new inequalities related to the rate of $L^{p}$ approximation by Cesàro means of the quadratic partial sums of double Vilenkin–Fourier series of functions from $L^{p}$.

## Introduction

Let $N_{+}$ denote the set of positive integers, and let $N:=N_{+} \cup \{0\}$. Let $m:= ( m_{0},m_{1},\ldots ) $ be a sequence of positive integers not less than 2. Denote by $Z_{m_{k}}:=\{0,1,\ldots,m _{k}-1\}$ the additive group of integers modulo $m_{k}$. Define the group $G_{m}$ as the complete direct product of the groups $Z_{m_{j}}$ with the product of the discrete topologies of $Z_{m_{j}}$.

The direct product of the measures

$$ \mu _{k} \bigl( \{j\} \bigr) :=\frac{1}{m_{k}} \quad ( j\in Z_{m_{k}} ) $$

is the Haar measure on $G_{m}$ with $\mu ( G_{m} ) =1$. If the sequence *m* is bounded, then $G_{m}$ is called a bounded Vilenkin group. In this paper, we consider only bounded Vilenkin groups. The elements of $G_{m}$ can be represented by sequences $x:= ( x_{0},x _{1},\ldots,x_{j},\ldots ) $ ($x_{j}\in Z_{m_{j}} $). The group operation + in $G_{m}$ is given by

$$ x+y= \bigl( ( x_{0}+y_{0} ) \operatorname{mod} m_{0},\ldots, ( x_{k}+y_{k} ) \operatorname{mod} m_{k},\ldots \bigr) $$

for $x:= ( x_{0},\ldots,x_{k},\ldots ) $ and $y:= ( y_{0},\ldots,y _{k},\ldots ) \in G_{m}$. The inverse of + will be denoted by −.

It is easy to give a base for the neighborhoods of $G_{m}$:

$$ \begin{gathered} I_{0} ( x ) :=G_{m}, \\ I_{n} ( x ) :=\{y\in G_{m}|y_{0}=x_{0}, \ldots,y _{n-1}=x_{n-1}\} \end{gathered} $$

for $x \in G_{m}$ and $n \in N$. Define $I_{n}:=I_{n} ( 0 ) $ for $n\in N_{+}$. Set $e_{n}:= ( 0,\ldots,0,1,0,\ldots ) \in G_{m}$, where the *n*th coordinate of which is 1, and the rest are zeros ($n\in N $).

We define the so-called generalized number system based on *m* as follows: $M_{0}:=1$, $M_{k+1}:=m_{k}M_{k}$ ($k\in N $). Then every $n \in N$ can be uniquely expressed as $n=\sum_{j=0} ^{\infty }n_{j}M_{j}$, where $n_{j} \in Z_{m_{j}}$ ($j \in N_{+} $), and only a finite number of $n_{j}$ differ from zero. We also use the following notation: $\vert n \vert :=\max \{k\in N:n_{k}\neq 0\}$ (i.e., $M_{|n|}\leq n< M_{|n|+1}$, $n\neq 0$). For $x \in G_{m}$, we denote $\vert x \vert :=\sum_{j=0}^{ \infty }\frac{x_{j}}{M_{j+1}}$ ($x_{j}\in Z_{m_{j}} $).

Next, we introduce on $G_{m}$ an orthonormal system, which is called the Vilenkin system. First, we define the complex-valued functions $r_{k} ( x ) :G_{m}\rightarrow C$, the generalized Rademacher functions, as follows:

$$ r_{k}(x):=\exp \frac{2\pi ix_{k}}{m_{k}} \quad \bigl( i^{2}=-1,x\in G_{m},k\in N \bigr) . $$

Now we define the Vilenkin system $\psi := ( \psi _{n}:n\in N ) $ on $G_{m}$ as

$$ \psi _{n} ( x ) :=\prod_{k=0}^{\infty }r_{k}^{n_{k}} ( x ) \quad ( n\in N ) . $$

In particular, if $m=2$, then we call this system the Walsh–Paley system. Each $\psi _{n}$ is a character of $G_{m}$, and all characters of $G_{m}$ are of this norm. Moreover, $\psi _{n} ( -x ) =\bar{ \psi }_{n} ( x ) $.

The Dirichlet kernels are defined by

$$ D_{n}:=\sum_{k=0}^{n-1}\psi _{k} \quad ( n\in N_{+} ) . $$

Recall that (see [20] or [23])

1 $$ D_{M_{n}} ( x ) = \textstyle\begin{cases} M_{n}& \text{if }x \in I_{n}, \\ 0&\text{if }x\notin I_{n}. \end{cases} $$

The Vilenkin system is orthonormal and complete in $L^{1} ( G _{m} ) $ (see [1]).

Next, we introduce some notation from the theory of two-dimensional Vilenkin system. Let *m̃* be a sequence like *m*. The relation between the sequences $( \tilde{m}_{n} ) $ and $( \tilde{M} _{n}) $ is the same as between sequences $( m_{n} ) $ and $(M_{n}) $. The group $G_{m}\times G_{\tilde{m}}$ is called a two-dimensional Vilenkin group. The normalized Haar measure is denoted by *μ* as in the one-dimensional case. We also suppose that $m=\tilde{m}$ and $G_{m}\times G_{\tilde{m}}=G_{m}^{2}$.

The norm of the space $L^{p} ( G_{m}^{2} ) $ is defined by

$$ \Vert f \Vert _{p}:= \biggl( \int _{G_{m}^{2}} \bigl\vert f ( x,y ) \bigr\vert ^{p}\,d \mu ( x,y ) \biggr) ^{1/p} \quad ( 1\leq p< \infty ) . $$

Denote by $C ( G_{m}^{2} ) $ the class of continuous functions on the group $G_{m}^{2}$ endowed with the supremum norm.

For brevity in notation, we write $L^{\infty } ( G_{m}^{2} ) $ instead of $C ( G_{m}^{2} ) $.

The two-dimensional Fourier coefficients, the rectangular partial sums of the Fourier series, and the Dirichlet kernels with respect to the two-dimensional Vilenkin system are defined as follows:

$$ \begin{gathered} \widehat{f} ( n_{1},n_{2} ) := \int _{G_{m}^{2}}f ( x,y ) \bar{\psi }_{n_{1}} ( x ) \bar{\psi }_{n_{2}} ( y ) \,d\mu ( x,y ) , \\ S_{n_{1},n_{2}} ( x,y,f ) :=\sum_{k_{1}=0}^{n_{1}-1} \sum_{k_{2}=0}^{n_{2}-1}\widehat{f} ( k_{1},k_{2} ) \psi _{k_{1}} ( x ) \psi _{k_{2}} ( y ) , \\ D_{n_{1},n_{2}} ( x,y ) :=D_{n_{1}} ( x ) D_{n_{2}} ( y ) , \end{gathered} $$

Denote

$$ \begin{gathered} S_{n}^{ ( 1 ) } ( x,y,f ) :=\sum _{l=0}^{n-1} \widehat{f} ( l,y ) \bar{\psi }_{l} ( x ) , \\ S_{m}^{ ( 2 ) } ( x,y,f ) :=\sum_{r=0}^{m-1} \widehat{f} ( x,r ) \bar{\psi }_{r} ( y ) , \end{gathered} $$

where

$$ \widehat{f} ( l,y ) = \int _{G_{m}}f ( x,y ) \psi _{l} ( x ) \,d\mu ( x ) $$

and

$$ \widehat{f} ( x,r ) = \int _{G_{m}}f ( x,y ) \psi _{r} ( y ) \,d\mu ( y ) . $$

The $(C,-\alpha )$ means of the double Vilenkin–Fourier series are defined as follows:

$$ \sigma _{n}^{-\alpha } ( f,x,y ) = \frac{1}{A_{n-1}^{-\alpha }}\sum _{j=1}^{n}A_{n-j}^{-\alpha -1}S _{j,j} ( f,x,y ) , $$

where

$$ A_{0}^{\alpha }=1, \qquad A_{n}^{\alpha }= \frac{ ( \alpha +1 ) \cdots ( \alpha +n ) }{n!}. $$

It is well known that (see [28])

2 $$\begin{aligned}& A_{n}^{\alpha }=\sum_{k=0}^{n}A_{k}^{\alpha -1}, \end{aligned}$$

3 $$\begin{aligned}& A_{n}^{\alpha }-A_{n-1}^{\alpha }=A_{n}^{\alpha -1}, \end{aligned}$$

and

4 $$ c_{1}(\alpha )n^{\alpha }\leq A_{n}^{\alpha }\leq c_{2} (\alpha )n ^{\alpha }, $$

where positive constants $c_{1}$ and $c_{2}$ depend on *α*.

The dyadic partial moduli of continuity of a function $f\in L^{p} ( G_{m}^{2} ) $ in the $L^{p}$-norm are defined by

$$ \omega _{1} \biggl( f,\frac{1}{M_{n}} \biggr) _{p}=\sup _{u\in I_{n}} \bigl\Vert f ( \cdot +u,\cdot ) -f ( \cdot ,\cdot ) \bigr\Vert _{p} $$

and

$$ \omega _{2} \biggl( f,\frac{1}{M_{n}} \biggr) _{p}=\sup _{v\in I_{n}} \bigl\Vert f ( \cdot ,\cdot +v ) -f ( \cdot ,\cdot ) \bigr\Vert _{p}, $$

whereas the dyadic mixed modulus of continuity is defined as follows:

$$ \begin{gathered} \omega _{1,2} \biggl( f,\frac{1}{M_{n}}, \frac{1}{M_{m}} \biggr) _{p} \\ \quad =\sup_{ ( u,v ) \in I_{n}\times I_{m}} \bigl\Vert f ( \cdot +u,\cdot +v ) -f ( \cdot +u,\cdot ) -f ( \cdot ,\cdot +v ) +f ( \cdot ,\cdot ) \bigr\Vert _{p}. \end{gathered} $$

It is clear that

$$ \omega _{1,2} \biggl( f,\frac{1}{M_{n}},\frac{1}{M_{m}} \biggr) _{p} \leq \omega _{1} \biggl( f,\frac{1}{M_{n}} \biggr) _{p}+\omega _{2} \biggl( f,\frac{1}{M _{m}} \biggr) _{p}. $$

The dyadic total modulus of continuity is defined by

$$ \omega \biggl( f,\frac{1}{M_{n}} \biggr) _{p}= \sup _{ ( u,v ) \in I_{n}\times I_{n}} \bigl\Vert f ( \cdot +u,\cdot +v ) -f ( \cdot ,\cdot ) \bigr\Vert _{p}. $$

The problems of summability of partial sums and Cesàro means for Walsh–Fourier series were studied in [2, 13–19, 21, 22, 25, 26].

The convergence issue of Fejér (and Cesàro) means on the Walsh and Vilenkin groups for unbounded case were studied in [3–11].

In his monograph [27], Zhizhinashvili investigated the behavior of Cesàro $(C, \alpha )$-means for double trigonometric Fourier series in detail. Goginava [18] studied the analogous question in the case of the Walsh system. In particular, the following theorems were proved.

### Theorem A

*Let* *f* *belong to* $L^{p} ( G_{2} ) $ *for some* $p \in [ 1,\infty ] $ *and* $\alpha \in ( 0,1 ) $. *Then*, *for any* $2^{k}\leq n<2^{k+1}$ ($k, n\in N$), *we have the inequality*

$$ \begin{aligned} \bigl\Vert \sigma _{2^{k}}^{-\alpha } ( f ) -f \bigr\Vert _{p}&\leq c ( \alpha ) \Biggl\{ 2^{k\alpha }\omega _{1} \bigl( f,1/2^{k-1} \bigr) _{p}+2^{k\alpha } \omega _{2} \bigl( f,1/2^{k-1} \bigr) _{p} \\ &\quad {} +\sum_{r=0}^{k-2}2^{r-k} \omega _{1} \bigl( f,1/2^{r} \bigr) _{p}+\sum _{s=0}^{k-2}2^{s-k}\omega _{2} \bigl( f,1/2^{s} \bigr) _{p} \Biggr\} . \end{aligned} $$

### Theorem B

*Let* *f* *belong to* $L^{p} ( G_{2} ) $ *for some* $p \in [ 1,\infty ] $ *and* $\alpha \in ( 0,1 ) $. *Then*, *for any* $2^{k}\leq n<2^{k+1}$ ($k, n\in N$), *we have the inequality*

$$ \begin{aligned} \bigl\Vert \sigma _{n}^{-\alpha } ( f ) -f \bigr\Vert _{p} &\leq c ( \alpha ) \Biggl\{ 2^{k\alpha }k\omega _{1} \bigl( f,1/2^{k-1} \bigr) _{p}+2^{k\alpha }k \omega _{2} \bigl( f,1/2^{k-1} \bigr) _{p} \\ &\quad {} {}+\sum_{r=0}^{k-2}2^{r-k} \omega _{1} \bigl( f,1/2^{r} \bigr) _{p}+\sum _{s=0}^{k-2}2^{s-k}\omega _{2} \bigl( f,1/2^{s} \bigr) _{p} \Biggr\} . \end{aligned} $$

In this paper, we state and prove analogous results in the case of double Vilenkin–Fourier series. Our main results are the following theorems.

### Theorem 1

*Let* *f* *belong to* $L^{p} ( G_{m}^{2} ) $ *for some* $p \in [ 1,\infty ] $ *and* $\alpha \in ( 0,1 ) $. *Then*, *for any* $M_{k}\leq n< M_{k+1}$ ($k, n\in N$), *we have the inequality*

$$ \begin{aligned} \bigl\Vert \sigma _{M_{k}}^{-\alpha } ( f ) -f \bigr\Vert _{p}&\leq c ( \alpha ) \Biggl( \omega _{1} ( f,1/M_{k-1} ) _{p}M_{k}^{\alpha }+\omega _{2} ( f,1/M_{l-1} ) _{p}M_{k}^{\alpha }\\ &\quad {} +\sum_{r=0}^{k-2} \frac{M_{r}}{M_{k}}\omega _{1} ( f,1/M _{r} ) _{p}+ \sum_{s=0}^{k-2}\frac{M_{s}}{M_{k}}\omega _{2} ( f,1/M_{s} ) _{p} \Biggr). \end{aligned} $$

### Theorem 2

*Let* *f* *belong to* $L^{p} ( G_{m}^{2} ) $ *for some* $p \in [ 1,\infty ] $ *and* $\alpha \in ( 0,1 ) $. *Then*, *for any* $M_{k}\leq n< M_{k+1}$ ($k, n\in N$), *we have the inequality*

$$ \begin{gathered} \bigl\Vert \sigma _{n}^{-\alpha } ( f ) -f \bigr\Vert _{p} \\ \quad \leq c ( \alpha ) \Biggl( \omega _{1} ( f,1/M_{k-1} ) _{p}M_{k}^{\alpha }\log n+\omega _{2} ( f,1/M_{l-1} ) _{p}M _{k}^{\alpha }\log n \\ \qquad {} +\sum_{r=0}^{k-2} \frac{M_{r}}{M_{k}}\omega _{1} ( f,1/M _{r} ) _{p}+ \sum_{s=0}^{k-2}\frac{M_{s}}{M_{k}}\omega _{2} ( f,1/M_{s} ) _{p} \Biggr). \end{gathered} $$

To make the proofs of these theorems clearer, we formulate some auxiliary lemmas in Sect. 2. Some of these lemmas are new and of independent interest. Detailed proofs can be found in Sect. 3.

## Auxiliary lemmas

To prove Theorems 1 and 2, we need the following three lemmas (see [1, 12], and [8], respectively)

### Lemma 1

*Let* $\alpha _{1}, \alpha _{2},\ldots,\alpha _{n}$ *be real numbers*. *Then*

$$ \frac{1}{n} \int _{G} \Biggl\vert \sum_{k=1}^{n} \alpha _{k}D _{k}(x) \Biggr\vert \,d\mu (x)\leq \frac{c}{\sqrt{n}} \Biggl( \sum_{k=1}^{n} \alpha _{k}^{2} \Biggr) ^{1/2}. $$

### Lemma 2

*Let* $\alpha _{1}, \alpha _{2},\ldots,\alpha _{n}$ *be real numbers*. *Then*

$$ \frac{1}{n} \int _{G_{m}^{2}} \Biggl\vert \sum_{k=1}^{n} \alpha _{k}D_{k} ( x ) D_{k} ( y ) \Biggr\vert \,d \mu ( x,y ) \leq \frac{c}{\sqrt{n}} \Biggl( \sum_{k=1} ^{n}\alpha _{k}^{2} \Biggr) ^{1/2}. $$

### Lemma 3

*Let* $0\leq j< n_{s}M_{s}$ *and* $0\leq n_{s}< m_{s}$. *Then*

$$ D_{n_{s}M_{s}-j}=D_{n_{s}M_{s}}-\psi _{n_{s}M_{s}-1}\bar{D}_{j}. $$

We also need the following new nemmas of independent interest.

### Lemma 4

*Let* *f* *belong to* $L^{p} ( G_{m}^{2} ) $ *for some* $p \in [ 1,\infty ] $. *Then*, *for every* $\alpha \in ( 0,1 ) $, *we have the inequality*

$$ \begin{aligned} I&:=\frac{1}{A_{n}^{-\alpha }} \Biggl\Vert \int _{G_{m}^{2}} \sum_{i=1}^{M_{k-1}}A_{n-i}^{-\alpha -1}D_{i} ( u ) D _{i} ( v ) \bigl[ f ( \cdot -u,\cdot - ) -f ( \cdot ,\cdot ) \bigr] \,d\mu (u,v) \Biggr\Vert _{p} \\ & \leq \sum_{r=0}^{k-2} \frac{M_{r}}{M_{k}}\omega _{1} ( f,1/M_{r} ) _{p}+ \sum_{s=0}^{k-2}\frac{M_{s}}{M _{k}}\omega _{2} ( f,1/M_{s} ) _{p} , \end{aligned} $$

*where* $M_{k}\leq n< M_{k+1}$.

### Lemma 5

*Let* $\alpha \in ( 0,1 ) $ *and* $p=M_{k},M_{k}+1,\ldots $ . *Then*

$$ \mathit{II}:= \int _{G_{m}^{2}} \Biggl\vert \sum_{i=1}^{M_{k}}A_{p-i} ^{-\alpha -1}D_{i} ( u ) D_{i} ( v ) \Biggr\vert \,d \mu (u,v)\leq c ( \alpha ) < \infty ,\quad k=1,2,\ldots . $$

### Lemma 6

*We have the inequality*

$$ \mathit{III}:= \int _{G_{m}^{2}} \Biggl\vert \sum_{i=1}^{n}A_{n-i} ^{-\alpha -1}D_{i} ( u ) D_{i} ( v ) \Biggr\vert \,d \mu (u,v)\leq c ( \alpha ) \log n $$

## The detailed proofs

### Proof of Lemma 3

Applying Abel’s transformation, from (2) we get

5 $$ \begin{aligned}[b] I&\leq \frac{1}{A_{n}^{-\alpha }} \Biggl\Vert \int _{G_{m}^{2}} \sum_{i=1}^{M_{k-1}-1}A_{n-i}^{-\alpha -2} \sum_{l=1} ^{i}D_{i} ( u ) D_{i} ( v ) \bigl[ f ( \cdot -u, \cdot -v ) -f ( \cdot ,\cdot ) \bigr] \,d\mu (u,v) \Biggr\Vert _{p} \\ &\quad {}+\frac{1}{A_{n}^{-\alpha }} \Biggl\Vert \int _{G_{m}^{2}}A_{n-M _{k-1}}^{-\alpha -1}\sum _{i=1}^{M_{k-1}}D_{i} ( u ) D _{i} ( v ) \bigl[ f ( \cdot -u,\cdot -v ) -f ( \cdot ,\cdot ) \bigr] \,d\mu (u,v) \Biggr\Vert _{p} \\ &:=I_{1}+I_{2}, \end{aligned} $$

where the first and second terms on the right side of inequality (5) are denoted by $I_{1}$ and $I_{2}$, respectively.

For $I_{2}$, we have the estimate

6 $$ \begin{aligned}[b] I_{2}&\leq \frac{1}{A_{n}^{-\alpha }} \Biggl\Vert \int _{G_{m} ^{2}}A_{n-M_{k-1}}^{-\alpha -1}\sum _{r=1}^{k-2}\sum_{i=M_{r}}^{M_{r+1}-1}D_{i} (u ) D_{i} ( v ) \\ &\quad {}\times \bigl[ f ( \cdot -u,\cdot -v ) -f ( \cdot ,\cdot ) \bigr] \Biggr\Vert _{p}\,d\mu (u,v) \\ &\leq \frac{1}{A_{n}^{-\alpha }} \Biggl\Vert \int _{G_{m}^{2}}A _{n-M_{k-1}}^{-\alpha -1}\sum _{r=1}^{k-2}\sum_{i=M_{r}} ^{M_{r+1}-1}D_{i} (u ) D_{i} ( v ) \\ &\quad {}\times \bigl[ f ( \cdot -u,\cdot -v ) -S_{M_{r},M_{r}} ( \cdot -u,\cdot -v,f ) \bigr] \,d\mu (u,v) \Biggr\Vert _{p} \\ &\quad {}+\frac{1}{A_{n}^{-\alpha }} \Biggl\Vert \int _{G_{m}^{2}}A_{n-M _{k-1}}^{-\alpha -1}\sum _{r=1}^{k-2}\sum_{i=M_{r}}^{M _{r+1}-1}D_{i} ( u ) D_{i} ( v ) \\ &\quad {}\times \bigl[ S_{M_{r},M_{r}} ( \cdot -u,\cdot -v,f ) -S_{M_{r},M_{r}} ( \cdot ,\cdot ,f ) \bigr] \,d\mu (u,v) \Biggr\Vert _{p} \\ &\quad {}+\frac{1}{A_{n}^{-\alpha }} \Biggl\Vert \int _{G_{m}^{2}}A_{n-M _{k-1}}^{-\alpha -1}\sum _{r=1}^{k-2}\sum_{i=M_{r}}^{M _{r+1}-1}D_{i} ( u ) D_{i} ( v ) \\ &\quad {}\times \bigl[ S_{M_{r},M_{r}} ( \cdot ,\cdot ,f ) -f ( \cdot ,\cdot ) \bigr] \,d\mu (u,v) \Biggr\Vert _{p}\\ &:=I_{21}+I_{22}+I_{23}, \end{aligned} $$

where the first, second, and third terms on the right side of inequality (6) are denoted by $I_{21}$, $I_{22}$, and $I_{23}$, respectively.

It is evident that

$$ \begin{aligned}[b] & \int _{G_{m}^{2}}\sum_{i=M_{r}}^{M_{r+1}-1}D_{i} ( u ) D_{i} ( v ) \bigl[ S_{M_{r},M_{r}} ( \cdot -u, \cdot -v,f ) -S_{M_{r},M_{r}} ( \cdot ,\cdot ,f ) \bigr] \,d\mu (u,v) \\ &\quad =\sum_{i=M_{r}}^{M_{r+1}-1} \biggl( \int _{G_{m}^{2}}D_{i} ( u ) D_{i} ( v ) S_{M_{r},M_{r}} ( \cdot -u, \cdot -v,f ) \,d\mu (u,v)-S_{M_{r},M_{r}} ( \cdot , \cdot ,f ) \biggr) \\ &\quad =\sum_{i=M_{r}}^{M_{r+1}-1} \bigl( S_{i} \bigl( \cdot ,\cdot ,S _{M_{r},M_{r}} ( f ) \bigr) -S_{M_{r},M_{r}} ( \cdot , \cdot ,f ) \bigr) \\ &\quad =\sum_{i=M_{r}}^{M_{r+1}-1} \bigl( S_{M_{r},M_{r}} ( \cdot ,\cdot ,f ) -S_{M_{r},M_{r}} ( \cdot ,\cdot ,f ) \bigr) =0. \end{aligned} $$

Hence

7 $$ I_{22}=0. $$

Moreover, by the generalized Minkowski inequality, Lemma 2, and by (1) and (4) we obtain

8 $$ \begin{aligned}[b] I_{21}&\leq \frac{1}{A_{n}^{-\alpha }} \bigl\vert A_{n-M_{k-1}}^{- \alpha -1} \bigr\vert \sum _{r=1}^{k-2} \int _{G_{m}^{2}} \Biggl\vert \sum_{i=M_{r}}^{M_{r+1}-1}D_{i} ( u ) D _{i} ( v ) \Biggr\vert \\ &\quad {}\times \bigl\Vert f ( \cdot -u,\cdot -v ) -S_{M_{r},M_{r}} ( \cdot -u,\cdot -v,f ) \bigr\Vert _{p}\,d\mu (u,v) \\ &\leq \frac{c ( \alpha ) }{M_{k}}\sum_{r=1}^{k-2} \bigl( \omega _{1} ( f,1/M_{r} ) _{p}+\omega _{2} ( f,1/M _{r} ) _{p} \bigr) \\ &\quad {}\times \int _{G_{m}^{2}} \Biggl\vert \sum_{i=M_{r}}^{M_{r+1}-1}D _{i} ( x ) D_{i} ( y ) \Biggr\vert \,d\mu (u,v) \\ &\leq c ( \alpha ) \sum_{r=1}^{k-2} \frac{M_{r}}{M_{k}} \bigl( \omega _{1} ( f,1/M_{r} ) _{p}+ \omega _{2} ( f,1/M_{r} ) _{p} \bigr) . \end{aligned} $$

The estimation of $I_{23}$ is analogous to that of $I_{21}$:

9 $$ I_{23}\leq c ( \alpha ) \sum_{r=1}^{k-2} \frac{M_{r}}{M _{k}} \bigl( \omega _{1} ( f,1/M_{r} ) _{p}+\omega _{2} ( f,1/M _{r} ) _{p} \bigr) . $$

Analogously, we can estimate $I_{1}$ as follows:

10 $$\begin{aligned} I_{1}&\leq \frac{1}{A_{n}^{-\alpha }}\sum _{r=1}^{k-2} \Biggl\Vert \int _{G_{m}^{2}}\sum_{i=M_{r}}^{M_{r+1}-1}A_{n-i} ^{-\alpha -2}\sum_{l=1}^{i}D_{l} ( u ) D_{l} ( v ) \\ &\quad {}\times \bigl[ f ( \cdot -u,\cdot -v ) -S_{M_{r},M_{r}} ( \cdot -u,\cdot -v,f ) \bigr] \,d\mu (u,v) \Biggr\Vert _{p} \\ &\quad {}+\frac{1}{A_{n}^{-\alpha }}\sum_{r=1}^{k-2} \Biggl\Vert \int _{G_{m}^{2}}\sum_{i=M_{r}}^{M_{r+1}-1}A_{n-i}^{- \alpha -2} \sum_{l=1}^{i}D_{l} ( u ) D_{l} ( v ) \\ &\quad {}\times \bigl[ S_{M_{r},M_{r}} ( \cdot -u,\cdot -v,f ) -S_{M _{r},M_{r}} ( \cdot ,\cdot ,f ) \bigr] \Biggr\Vert _{p}\,d \mu (u,v) \\ &\quad {}+\frac{1}{A_{n}^{-\alpha }}\sum_{r=1}^{k-2} \Biggl\Vert \int _{G_{m}^{2}}\sum_{i=M_{r}}^{M_{r+1}-1}A_{n-i}^{- \alpha -2} \sum_{l=1}^{i}D_{l} ( u ) D_{l} ( v ) \\ &\quad {}\times \bigl[ S_{M_{r},M_{r}} ( \cdot ,\cdot ,f ) -f ( \cdot ,\cdot ) \bigr] \,d\mu (u,v) \Biggr\Vert _{p} \\ &\leq \frac{1}{A_{n}^{-\alpha }}\sum_{r=1}^{k-2} \int _{G_{m}^{2}} \Biggl\vert \sum_{i=M_{r}}^{M_{r+1}-1}A_{n-i} ^{-\alpha -2}\sum_{l=1}^{i}D_{l} ( u ) D_{l} ( v ) \Biggr\vert \\ &\quad {}\times \bigl\Vert f ( \cdot -u,\cdot -v ) -S_{M_{r},M_{r}} ( \cdot -u,\cdot -v,f ) \bigr\Vert _{p}\,d\mu (u,v) \\ &\quad {}+\frac{1}{A_{n}^{-\alpha }}\sum_{r=1}^{k-2} \int _{G_{m} ^{2}} \Biggl\vert \sum_{i=M_{r}}^{M_{r+1}-1}A_{n-i}^{-\alpha -2} \sum_{l=1}^{i}D_{l} ( u ) D_{l} ( v ) \Biggr\vert \\ &\quad {}\times \bigl\Vert S_{M_{r},M_{r}} ( \cdot ,\cdot ,f ) -f ( \cdot ,\cdot ) \bigr\Vert _{p} \,d\mu (u,v) \\ &\leq c ( \alpha ) M_{k}^{\alpha }\sum _{r=1}^{k-2} \sum_{i=M_{r}}^{M_{r+1}-1} ( n-i ) ^{-\alpha -2}i \bigl( \omega _{1} ( f,1/M_{r} ) _{p}+\omega _{2} ( f,1/M _{r} ) _{p} \bigr) \\ &\leq c ( \alpha ) M_{k}^{\alpha }\sum _{r=1}^{k-2} \sum_{i=M_{r}}^{M_{r+1}-1} ( n-M_{r+1}-1 ) ^{-\alpha -2}i \bigl( \omega _{1} ( f,1/M_{r} ) _{p}+\omega _{2} ( f,1/M _{r} ) _{p} \bigr) \\ &\leq c ( \alpha ) \sum_{r=0}^{k-2} \frac{M_{r}}{M_{k}} \bigl( \omega _{1} ( f,1/M_{r} ) _{p}+ \omega _{2} ( f,1/M_{r} ) _{p} \bigr) . \end{aligned}$$

By combining (7)–(9) with (10) for *I* we find that

11 $$ I\leq c ( \alpha ) \sum_{r=0}^{k-2} \frac{M_{r}}{M _{k}} \bigl( \omega _{1} ( f,1/M_{r} ) _{p}+\omega _{2} ( f,1/M _{r} ) _{p} \bigr) . $$

The proof of Lemma 3 is complete. □

### Proof of Lemma 4

It is evident that

12 $$ \begin{aligned}[b] \mathit{II}&\leq \int _{G_{m}^{2}} \Biggl\vert \sum_{i=1}^{M_{k}-1}A _{p-M_{k}+i}^{-\alpha -1}D_{M_{k}-i} ( u ) D_{M_{k}-i} ( v ) \Biggr\vert \,d\mu (u,v) \\ &\quad {}+ \bigl\vert A_{p-M_{k}}^{-\alpha -1} \bigr\vert \int _{G_{m} ^{2}}D_{M_{k}} ( u ) D_{M_{k}} ( v ) \,d\mu (u,v)\\ &:=\mathit{II} _{1}+\mathit{II}_{2}, \end{aligned} $$

where the first and second terms on the right side of inequality (12) are denoted by $\mathit{II}_{1}$ and $\mathit{II}_{2}$, respectively.

From (1) by $\vert A_{p-M_{k}}^{-\alpha -1} \vert \leq 1$ we get that

13 $$ \mathit{II}_{2}\leq 1. $$

Moreover, by Lemma 3 we have that

14 $$\begin{aligned} \mathit{II}_{1}&\leq \int _{G_{m}^{2}} \Biggl\vert \sum_{i=1}^{M_{k}-1}A _{p-M_{k}+i}^{-\alpha -1}\bar{D}_{i} ( u ) \bar{D}_{i} ( v ) \Biggr\vert \,d\mu (u,v) \\ &\quad {}+ \int _{G_{m}^{2}}D_{M_{k}} ( u ) \Biggl\vert \sum _{i=1}^{M_{k}-1}A_{p-M_{k}+i}^{-\alpha -1} \bar{D}_{i} ( v ) \Biggr\vert \,d\mu (u,v) \\ &\quad {}+ \int _{G_{m}^{2}}D_{M_{k}} ( v ) \Biggl\vert \sum _{i=1}^{M_{k}-1}A_{p-M_{k}+i}^{-\alpha -1} \bar{D}_{i} ( u ) \Biggr\vert \,d\mu (u,v) \\ &\quad {}+ \Biggl\vert \sum_{i=1}^{M_{k}-1}A_{p-M_{k}+i}^{-\alpha -1} \Biggr\vert \int _{G_{m}^{2}}D_{M_{k}} ( u ) D_{M_{k}} ( v ) \,d\mu (u,v) \\ &:=\mathit{II}_{11}+\mathit{II}_{12}+\mathit{II}_{13}+ \mathit{II}_{14}, \end{aligned}$$

where the first, second, third, and fourth terms on the right side of inequality (14) are denoted by $\mathit{II}_{11}$, $\mathit{II}_{12}$, $\mathit{II}_{13}$, and $\mathit{II}_{14}$ respectively.

From (1) and (4) it follows that

15 $$ \mathit{II}_{14}\leq c ( \alpha ) \sum_{v=1}^{\infty }v^{- \alpha -1}< \infty . $$

By Applying Abel’s transformation, in view of Lemma 2, we have that

16 $$ \begin{aligned}[b] \mathit{II}_{11}&\leq \int _{G_{m}^{2}} \Biggl\vert \sum_{i=1}^{M _{k}-2}A_{p-M_{k}+i}^{-\alpha -2} \sum_{l=1}^{i}\bar{D}_{l} ( u ) \bar{D}_{l} ( v ) \Biggr\vert \,d\mu (u,v) \\ &\quad {}+ \int _{G_{m}^{2}} \Biggl\vert A_{p-1}^{-\alpha -1}\sum _{i=1}^{M_{k}-1}\bar{D}_{i} ( u ) \bar{D}_{i} ( v ) \Biggr\vert \,d\mu (u,v) \\ &\leq c ( \alpha ) \Biggl\{ \sum_{v=1}^{M_{k}-2} ( p-M_{k}+i ) ^{-\alpha -2}i+ ( p-1 ) ^{-\alpha -1}M_{k} \Biggr\} \\ &\leq c ( \alpha ) \Biggl\{ \sum_{i=1}^{\infty }i^{- \alpha -1}+M_{k}^{-\alpha } \Biggr\} < \infty . \end{aligned} $$

The estimation of $\mathit{II}_{12}$ and $\mathit{II}_{13}$ are analogous to the estimation of $\mathit{II}_{11}$. Applying Abel’s transformation, in view of Lemma 1, we find that

17 $$ \begin{aligned}[b] \mathit{II}_{12}&\leq \int _{G_{m}^{2}}D_{M_{k}} ( u ) \Biggl\vert \sum _{i=1}^{M_{k}-2}A_{p-M_{k}+i}^{-\alpha -2}\sum _{l=1} ^{i}\bar{D}_{l} ( v ) \Biggr\vert \,d\mu (u,v) \\ &\quad {}+ \int _{G_{m}^{2}}D_{M_{k}} ( u ) \Biggl\vert A_{p-1} ^{-\alpha -1}\sum_{i=1}^{M_{k}-1} \bar{D}_{i} ( v ) \Biggr\vert \,d \mu (u,v) \\ &\leq c ( \alpha ) \Biggl\{ \sum_{v=1}^{M_{k}-2} ( p-M_{k}+i ) ^{-\alpha -2}i+ ( p-1 ) ^{-\alpha -1}M_{k} \Biggr\} \\ &\leq c ( \alpha ) \Biggl\{ \sum_{i=1}^{\infty }i^{- \alpha -1}+M_{k}^{-\alpha } \Biggr\} < \infty \end{aligned} $$

and

18 $$\begin{aligned} \mathit{III}_{12}&\leq \int _{G_{m}^{2}}D_{M_{k}} ( v ) \Biggl\vert \sum _{i=1}^{M_{k}-2}A_{p-M_{k}+i}^{-\alpha -2}\sum _{l=1} ^{i}\bar{D}_{l} ( u ) \Biggr\vert \,d\mu (u,v) \\ &\quad {}+ \int _{G_{m}^{2}}D_{M_{k}} ( v ) \Biggl\vert A_{p-1} ^{-\alpha -1}\sum_{i=1}^{M_{k}-1} \bar{D}_{i} ( u ) \Biggr\vert \,d \mu (u,v) \\ &\leq c ( \alpha ) \Biggl\{ \sum_{v=1}^{M_{k}-2} ( p-M_{k}+i ) ^{-\alpha -2}i+ ( p-1 ) ^{-\alpha -1}M_{k} \Biggr\} \\ &\leq c ( \alpha ) \Biggl\{ \sum_{i=1}^{\infty }i^{- \alpha -1}+M_{k}^{-\alpha } \Biggr\} < \infty . \end{aligned}$$

The proof is complete by combining (12)–(18). □

### Proof of Lemma 5

Let

$$ n=n_{k_{1}}M_{k_{1}}+\cdots+n_{k_{s}}M_{k_{s}}, \quad k_{1}>\cdots >k_{s} \geq 0. $$

Denote

$$ n^{ ( i ) }=n_{k_{i}}M_{k_{i}}+\cdots +n_{k_{s}}M_{k_{s}}, \quad i=1,2,\ldots,s. $$

Since (see [20])

19 $$ D_{j+n_{A}M_{A}}=D_{n_{A}M_{A}}+\psi _{n_{A}M_{A}}D_{j}, $$

we find that

20 $$ \begin{aligned}[b] \mathit{III}&\leq \int _{G_{m}^{2}} \Biggl\vert \sum_{i=1}^{n_{k_{1}}M _{k_{1}}}A_{n-i}^{-\alpha -1}D_{i} ( u ) D_{i} ( v ) \Biggr\vert \,d\mu (u,v) \\ &\quad {}+ \int _{G_{m}^{2}} \Biggl\vert \sum_{i=1}^{n^{ ( 2 ) }}A_{n^{ ( 2 ) }-i}^{-\alpha -1}D_{i} ( u ) D_{i} ( v ) \Biggr\vert \,d\mu (u,v) \\ &\quad {}+ \int _{G_{m}^{2}}D_{n_{k_{1}}M_{k_{1}}} ( u ) D_{n _{k_{1}}M_{k_{1}}} ( v ) \Biggl\vert \sum_{i=1}^{n^{ ( 2 ) }}A_{n^{ ( 2 ) }-i}^{-\alpha -1} \Biggr\vert \,d \mu (u,v) \\ &\quad {}+ \int _{G_{m}^{2}}D_{n_{k_{1}}M_{k_{1}}} ( u ) \Biggl\vert \sum _{i=1}^{n^{ ( 2 ) }}A_{n^{ ( 2 ) }-i}^{- \alpha -1}D_{i} ( v ) \Biggr\vert \,d\mu (u,v) \\ &\quad {}+ \int _{G_{m}^{2}}D_{n_{k_{1}}M_{k_{1}}} ( v ) \Biggl\vert \sum _{i=1}^{n^{ ( 2 ) }}A_{n^{ ( 2 ) }-i}^{- \alpha -1}D_{i} ( u ) \Biggr\vert \,d\mu (u,v) \\ &:=\mathit{III}_{1}+\mathit{III}_{2}+\mathit{III}_{3}+ \mathit{III}_{4}+\mathit{III}_{5}, \end{aligned} $$

where the first, second, third, fourth, and fifth terms on the right side of inequality (20) are denoted by $\mathit{III}_{1}$, $\mathit{III}_{2}$, $\mathit{III}_{3}$, $\mathit{III}_{4}$, and $\mathit{III}_{5}$, respectively.

By (1) we have that

21 $$ \mathit{III}_{3}\leq c ( \alpha ) . $$

Moreover, since (see [24])

22 $$ \Biggl\vert \sum_{i=1}^{n}A_{n-i}^{-\alpha -1}D_{i} ( u ) \Biggr\vert =O \bigl( \vert u \vert ^{\alpha -1} \bigr) , $$

for $\mathit{III}_{4}$, we get that

23 $$ \begin{aligned}[b] \mathit{III}_{4}&\leq \int _{G_{m}^{2}}D_{n_{k_{1}}M_{k_{1}}} ( u ) \vert v \vert ^{\alpha -1}\,d\mu (u,v) \\ &\leq \int _{G_{m}} \vert v \vert ^{\alpha -1}\,d\mu ( v ) = \frac{1}{ \alpha }< \infty . \end{aligned} $$

Analogously, we find that

24 $$ \begin{aligned}[b] \mathit{III}_{5}&\leq \int _{G_{m}^{2}}D_{n_{k_{1}}M_{k_{1}}} ( v ) \vert u \vert ^{\alpha -1}\,d\mu (u,v) \\ &\leq \int _{G_{m}} \vert u \vert ^{\alpha -1}\,d\mu ( v ) = \frac{1}{ \alpha }< \infty . \end{aligned} $$

For $r\in \{0,\ldots m_{A}-1\}$ and $0\leq j< M_{A}$ (see [20]), this yields that

$$ D_{j+rM_{A}}= \Biggl( \sum_{q=0}^{r-1} \psi _{M_{A}}^{q} \Biggr) D _{M_{A}}+\psi _{M_{A}}^{r}D_{j}. $$

Thus we have

$$ \begin{gathered} \int _{G_{m}^{2}}\sum_{i=1}^{n_{k_{1}}M_{k_{1}}-1}A_{n-i} ^{-\alpha -1}D_{i} ( u ) D_{i} ( v ) \,d\mu (u,v) \\ \quad \leq \int _{G_{m}^{2}}\sum_{r=0}^{n_{k_{1}}-1} \sum_{i=0}^{M_{k_{1}}-1}A_{n-i-rM_{k_{1}}}^{-\alpha -1}D_{i+rM_{k _{1}}} ( u ) D_{i+rM_{k_{1}}} ( v ) \,d\mu (u,v) \\ \quad \leq \int _{G_{m}^{2}}\sum_{r=0}^{n_{k_{1}}-1} \sum_{i=0}^{M_{k_{1}}-1}A_{n-i-rM_{k_{1}}}^{-\alpha -1} \Biggl( \sum_{q=0}^{r-1}\psi _{M_{k_{1}}}^{q} \Biggr) D_{M_{k_{1}}} ( u ) \\ \qquad {}\times \Biggl( \sum_{q=0}^{r-1} \psi _{M_{k_{1}}}^{q} \Biggr) D _{M_{k_{1}}} ( v ) \,d\mu (u,v) \\ \qquad {}+ \int _{G_{m}^{2}}\sum_{r=0}^{n_{k_{1}}-1} \sum_{i=0}^{M_{k_{1}}-1}A_{n-i-rM_{k_{1}}}^{-\alpha -1} \Biggl( \sum_{q=0}^{r-1}\psi _{M_{k_{1}}}^{q} \Biggr) D_{M_{k_{1}}} ( u ) \psi _{M_{A}}^{r}D_{i} ( v ) \,d\mu (u,v) \\ \qquad {}+ \int _{G_{m}^{2}}\sum_{r=0}^{n_{k_{1}}-1} \sum_{i=0}^{M_{k_{1}}-1}A_{n-i-rM_{k_{1}}}^{-\alpha -1} \psi _{M_{A}}^{r}D _{i} ( u ) \Biggl( \sum _{q=0}^{r-1}\psi _{M_{k_{1}}} ^{q} \Biggr) D_{M_{k_{1}}} ( v ) \,d\mu (u,v) \\ \qquad {}+ \int _{G_{m}^{2}}\sum_{r=0}^{n_{k_{1}}-1} \sum_{i=0}^{M_{k_{1}}-1}A_{n-i-rM_{k_{1}}}^{-\alpha -1} \psi _{M_{A}}^{r}D _{i} ( u ) \psi _{M_{A}}^{r}D_{i} ( v ) \,d\mu (u,v). \end{gathered} $$

On the other hand, by (1) and (4) we obtain that

$$ \int _{G_{m}^{2}}A_{n-n_{k_{1}}M_{k_{1}}}^{-\alpha -1}D_{n_{k _{1}}M_{k_{1}}} ( u ) D_{n_{k_{1}}M_{k_{1}}} ( v ) \,d \mu (u,v)\leq c ( \alpha ). $$

Consequently, for $\mathit{III}_{1}$, we have the estimate

25 $$ \begin{aligned}[b] \mathit{III}_{1}&\leq \int _{G_{m}^{2}}D_{M_{k_{1}}} ( u ) D_{M _{k_{1}}} ( v ) \Biggl\vert \sum_{r=0}^{n_{k_{1}}-1} \sum _{i=1}^{M_{k_{1}}}A_{n-i-rM_{k_{1}}}^{-\alpha -1} \Biggr\vert \,d \mu (u,v) \\ &\quad {}+ \int _{G_{m}^{2}}D_{M_{k_{1}}} ( u ) \Biggl\vert \sum _{r=0}^{n_{k_{1}}-1}\sum_{i=1}^{M_{k_{1}}}A_{n-i-rM_{k _{1}}}^{-\alpha -1}D_{i} ( v ) \Biggr\vert \,d\mu (u,v) \\ &\quad {}+ \int _{G_{m}^{2}}D_{M_{k_{1}}} ( v ) \Biggl\vert \sum _{r=0}^{n_{k_{1}}-1}\sum_{i=1}^{M_{k_{1}}}A_{n-i-rM_{k _{1}}}^{-\alpha -1}D_{i} ( u ) \Biggr\vert \,d\mu (u,v) \\ &\quad {}+ \int _{G_{m}^{2}} \Biggl\vert \sum_{r=0}^{n_{k_{1}}-1} \sum_{i=1}^{M_{k_{1}}}A_{n-i-rM_{k_{1}}}^{-\alpha -1}D_{i} ( u ) D_{i} ( v ) \Biggr\vert \,d\mu (u,v)+c ( \alpha ) \\ &:=\mathit{III}_{11}+\mathit{III}_{12}+ \mathit{III}_{13}+\mathit{III}_{14}+c ( \alpha ) , \end{aligned} $$

where the first, second, third, and fourth terms on the right side of inequality (25) are denoted by $\mathit{III}_{11}$, $\mathit{III}_{12} $, $\mathit{III}_{13}$, and $\mathit{III}_{14}$, respectively.

From Lemma 4 we have that

26 $$ \mathit{III}_{14}\leq c ( \alpha ) . $$

The estimation of $\mathit{III}_{11}$ is analogous to that of $\mathit{III}_{3}$, and we find that

27 $$ \mathit{III}_{11}\leq c ( \alpha ) . $$

The estimation of $\mathit{III}_{12}$ and $\mathit{III}_{13}$ is analogous to that of $\mathit{III}_{4}$, and we obtain that

28 $$ \mathit{III}_{12}< \infty $$

and

29 $$ \mathit{III}_{13}< \infty . $$

After substituting (21) and (23)–(29) into (20), we conclude that

$$ \begin{gathered} \int _{G_{m}^{2}} \Biggl\vert \sum_{i=1}^{n}A_{n-i}^{- \alpha -1}D_{i} ( u ) D_{i} ( v ) \Biggr\vert \,d \mu (u,v) \\ \quad \leq \int _{G_{m}^{2}} \Biggl\vert \sum_{i=1}^{n^{ ( 2 ) }}A_{n^{ ( 2 ) }-i}^{-\alpha -1}D_{i} ( u ) D_{i} ( v ) \Biggr\vert \,d\mu (u,v)+c ( \alpha ) \\ \quad \leq \cdots\leq \int _{G_{m}^{2}} \Biggl\vert \sum_{i=1}^{n ^{ ( s ) }}A_{n^{ ( s ) }-i}^{-\alpha -1}D_{i} ( u ) D_{i} ( v ) \Biggr\vert \,d\mu (u,v)+c ( \alpha ) s \\ \quad \leq c ( \alpha ) +c ( \alpha ) s\leq c ( \alpha ) \log n. \end{gathered} $$

The proof is complete. □

Now we are ready to prove the main results.

### Proof of Theorem 1

It is evident that

30 $$ \begin{gathered}[b] \bigl\Vert \sigma _{M_{k}}^{-\alpha } ( f ) -f \bigr\Vert _{p} \\ \quad \leq \frac{1}{A_{M_{k}-1}^{-\alpha }} \Biggl\Vert \int _{G_{m} ^{2}}\sum_{i=1}^{M_{k-1}}A_{M_{k}-i}^{-\alpha -1}D_{i} ( u ) D_{i} ( v ) \bigl[ f ( \cdot -u,\cdot -v ) -f ( \cdot ,\cdot ) \bigr] \,d\mu (u,v) \Biggr\Vert _{p} \\ \qquad {}+\frac{1}{A_{M_{k}-1}^{-\alpha }} \Biggl\Vert \int _{G_{m}^{2}} \sum_{i=M_{k-1}+1}^{M_{k}}A_{M_{k}-i}^{-\alpha -1}D_{i} ( u ) D_{i} ( v ) \bigl[ f ( \cdot -u,\cdot -v ) -f ( \cdot ,\cdot ) \bigr] \,d\mu (u,v) \Biggr\Vert _{p} \\ \quad :=I+\mathit{II}. \end{gathered} $$

From Lemma 5 it follows that

31 $$ I\leq c ( \alpha ) \sum_{r=0}^{k-2} \frac{M_{r}}{M _{k}} \bigl( \omega _{1} ( f,1/M_{r} ) _{p}+\omega _{2} ( f,1/M _{r} ) _{p} \bigr) . $$

Moreover, for *II*, we have the estimate

32 $$ \begin{aligned}[b] \mathit{II}&\leq \frac{1}{A_{M_{k}-1}^{-\alpha }} \Biggl\Vert \int _{G_{m}^{2}}\sum_{i=M_{k-1}+1}^{M_{k}}A_{M_{k}-i}^{- \alpha -1}D_{i} ( u ) D_{i} ( v ) \\ &\quad {}\times \bigl[ f ( \cdot -u,\cdot -v ) -S_{M_{k-1}} ^{ ( 1 ) } ( \cdot -u,\cdot -v,f ) \bigr] \,d\mu (u,v) \Biggr\Vert _{p} \\ &\quad {}+\frac{1}{A_{M_{k}-1}^{-\alpha }} \Biggl\Vert \int _{G_{m}^{2}} \sum_{i=M_{k-1}+1}^{M_{k}}A_{M_{k}-i}^{-\alpha -1}D_{i} ( u ) D_{i} ( v ) \\ &\quad {}\times \bigl[ S_{M_{k-1}}^{ ( 1 ) } ( \cdot -u, \cdot -v,f ) -f ( \cdot ,\cdot ) \bigr] \,d\mu (u,v) \Biggr\Vert _{p}\\ &:=\mathit{II}_{1}+\mathit{II}_{2}, \end{aligned} $$

where the first and second terms on the right side of inequality (32) are denoted by $\mathit{II}_{1}$ and $\mathit{II}_{2}$, respectively.

In view of the generalized Minkowski inequality, by (4) and Lemma 5 we get that

33 $$ \begin{aligned}[b] \mathit{II}_{1}&\leq \frac{1}{A_{M_{k}-1}^{-\alpha }} \int _{G_{m}^{2}} \Biggl\vert \sum_{i=M_{k-1}+1}^{M_{k}}A_{M_{k}-i}^{-\alpha -1}D _{i} ( u ) D_{i} ( v ) \Biggr\vert \\ &\quad {}\times \bigl\Vert f ( \cdot -u,\cdot -v ) -S_{M_{k-1}}^{ ( 1 ) } ( \cdot -u,\cdot -v,f ) \bigr\Vert _{p}\,d \mu (u,v) \\ &\leq c ( \alpha ) M_{k}^{\alpha }\omega _{1} ( f,1/M_{k-1} ) _{p}. \end{aligned} $$

The estimation of $\mathit{II}_{2}$ is analogous to that of $\mathit{II}_{1}$, and we find that

34 $$ \mathit{II}_{2}\leq c ( \alpha ) M_{k}^{\alpha }\omega _{2} ( f,1/M _{k-1} ) _{p}. $$

Combining (30)–(34), we obtain the proof of Theorem 1. □

### Proof of Theorem 2

It is evident that

35 $$ \begin{gathered}[b] \bigl\Vert \sigma _{n}^{-\alpha } ( f ) -f \bigr\Vert _{p} \\ \quad \leq \frac{1}{A_{n-1}^{-\alpha }} \Biggl\Vert \int _{G_{m}^{2}} \sum_{i=1}^{M_{k-1}}A_{n-i}^{-\alpha -1}D_{i} ( u ) D _{i} ( v ) \bigl[ f ( \cdot -u,\cdot -v ) -f ( \cdot ,\cdot ) \bigr] \,d\mu (u,v) \Biggr\Vert _{p} \\ \qquad {}+\frac{1}{A_{n-1}^{-\alpha }} \Biggl\Vert \int _{G_{m}^{2}} \sum_{i=M_{k-1}+1}^{M_{k}}A_{n-i}^{-\alpha -1}D_{i} ( u ) D_{i} ( v ) \bigl[ f ( \cdot -u,\cdot -v ) -f ( \cdot ,\cdot ) \bigr] \,d\mu (u,v) \Biggr\Vert _{p} \\ \qquad {}+\frac{1}{A_{n-1}^{-\alpha }} \Biggl\Vert \int _{G_{m}^{2}} \sum_{i=M_{k}+1}^{n}A_{n-i}^{-\alpha -1}D_{i} ( u ) D _{i} ( v ) \bigl[ f ( \cdot -u,\cdot -v ) -f ( \cdot ,\cdot ) \bigr] \,d\mu (u,v) \Biggr\Vert _{p} \\ \quad :=I+\mathit{II}+\mathit{III}, \end{gathered} $$

where the first, second, and third terms on the right side of inequality (35) are denoted by *I*, *II*, and *III*, respectively.

From Lemma 4 it follows that

36 $$ I\leq c ( \alpha ) \sum_{r=0}^{k-2} \frac{M_{r}}{M _{k}} \bigl( \omega _{1} ( f,1/M_{r} ) _{p}+\omega _{2} ( f,1/M _{r} ) _{p} \bigr) . $$

Next, we repeat the arguments just in the same way as in the proof of Theorem 1 and find that

37 $$ \mathit{II}\leq c ( \alpha ) M_{k}^{\alpha } \bigl( \omega _{1} ( f,1/M_{k-1} ) _{p}+\omega _{2} ( f,1/M_{k-1} ) _{p} \bigr) . $$

On the other hand, for III, we have

38 $$\begin{aligned} \mathit{III}&\leq \frac{1}{A_{n-1}^{-\alpha }} \Biggl\Vert \int _{G_{m} ^{2}}\sum_{i=M_{k}+1}^{n}A_{n-i}^{-\alpha -1}D_{i} ( u ) D_{i} ( v ) \\ &\quad {}\times \bigl[ f ( \cdot -u,\cdot -v ) -f ( \cdot ,\cdot ) \bigr] \Biggr\Vert _{p}\,d\mu (u,v) \\ &\leq \frac{1}{A_{n}^{-\alpha }} \Biggl\Vert \int _{G_{m}^{2}} \sum_{i=M_{k}+1}^{n}A_{n-i}^{-\alpha -1}D_{i} ( u ) D _{i} ( v ) \\ &\quad {}\times \bigl[ f ( \cdot -u,\cdot -v ) -S_{M_{k},M_{k}} ( \cdot -u,\cdot -v,f ) \bigr] \,d\mu (u,v) \Biggr\Vert _{p} \\ &\leq \frac{1}{A_{n}^{-\alpha }} \Biggl\Vert \int _{G_{m}^{2}} \sum_{i=M_{k}+1}^{n}A_{n-i}^{-\alpha -1}D_{i} ( u ) D _{i} ( v ) \\ &\quad {}\times \bigl[ S_{M_{k},M_{k}} ( \cdot -u,\cdot -v,f ) -S_{M_{k},M_{k}} ( \cdot ,\cdot ,f ) \bigr] \,d\mu (u,v) \Biggr\Vert _{p} \\ &\leq \frac{1}{A_{n}^{-\alpha }} \Biggl\Vert \int _{G_{m}^{2}} \sum_{i=M_{k}+1}^{n}A_{n-i}^{-\alpha -1}D_{i} ( u ) D _{i} ( v ) \\ &\quad {}\times \bigl[ S_{M_{k},M_{k}} ( \cdot ,\cdot ,f ) -f ( \cdot ,\cdot ) \bigr] \,d\mu (u,v) \Biggr\Vert _{p} \\ &:= \mathit{III}_{1}+\mathit{III}_{2}+\mathit{III}_{3}, \end{aligned}$$

where the first, second, and third terms on the right side of inequality (38) are denoted by $\mathit{III}_{1}$, $\mathit{III}_{2}$, and $\mathit{III}_{3}$, respectively.

It is easy to show that

39 $$ \mathit{III}_{2}=0. $$

By the generalized Minkowski inequality and Lemma 5, for $\mathit{III}_{1}$, we obtain that

40 $$ \begin{aligned}[b] \mathit{III}_{1}&\leq \frac{1}{A_{n}^{-\alpha }} \int _{G_{m}^{2}} \Biggl\vert \sum_{i=M_{k}+1}^{n}A_{n-i}^{-\alpha -1}D_{i} ( u ) D_{i} ( v ) \Biggr\vert \\ &\quad {}\times \bigl\Vert f ( \cdot -u,\cdot -v ) -S_{M_{r},M_{r}} ( \cdot -u,\cdot -v,f ) \bigr\Vert _{p}\,d\mu (u,v) \\ &\leq c ( \alpha ) M_{k}^{\alpha } \bigl( \omega _{1} ( f,1/M _{k-1} ) _{p}+\omega _{2} ( f,1/M_{k-1} ) _{p} \bigr) \\ &\quad {}\times \int _{G_{m}^{2}} \Biggl\vert \sum_{v=M_{k}+1}^{n}A _{n-v}^{-\alpha -1}D_{v} ( u ) D_{v} ( v ) \Biggr\vert \,d \mu (u,v) \\ &\leq c ( \alpha ) M_{k}^{\alpha }\log n \bigl( \omega _{1} ( f,1/M_{k-1} ) _{p}+\omega _{2} ( f,1/M_{k-1} ) _{p} \bigr) . \end{aligned} $$

The estimation of $\mathit{III}_{3}$ is analogous to that of $\mathit{III}_{2}$, and we find that

41 $$ \mathit{III}_{3}\leq c ( \alpha ) M_{k}^{\alpha } \log n \bigl( \omega _{1} ( f,1/M_{k-1} ) _{p}+\omega _{2} ( f,1/M_{k-1} ) _{p} \bigr) . $$

After substituting (36)–(37) and (41) into (35), we obtain the proof of Theorem 2. □
